# Effect of Oxidative Stress-Induced Apoptosis on Active FGF23 Levels in MLO-Y4 Cells: The Protective Role of 17-β-Estradiol

**DOI:** 10.3390/ijms23042103

**Published:** 2022-02-14

**Authors:** Vladana Domazetovic, Irene Falsetti, Simone Ciuffi, Teresa Iantomasi, Gemma Marcucci, Maria Teresa Vincenzini, Maria Luisa Brandi

**Affiliations:** 1 Department of Experimental and Clinical Biomedical Sciences, University of Florence, 50134 Florence, Italy; vladana.domazetovic@unifi.it (V.D.); irene.falsetti@unifi.it (I.F.); simone.ciuffi@unifi.it (S.C.); teresa.iantomasi@unifi.it (T.I.); gemma.marcucci@unifi.it (G.M.); mariateresa.vincenzini@unifi.it (M.T.V.); 2 Fondazione Italiana Ricerca sulle Malattie dell’Osso (FIRMO Onlus), 50141 Florence, Italy

**Keywords:** FGF23, oxidative stress-induced apoptosis, estrogen

## Abstract

The discovery that osteocytes secrete phosphaturic fibroblast growth factor 23 (FGF23) has defined bone as an endocrine organ. However, the autocrine and paracrine functions of FGF23 are still unknown. The present study focuses on the cellular and molecular mechanisms involved in the complex control of FGF23 production and local bone remodeling functions. FGF23 was assayed using ELISA kit in the presence or absence of 17β–estradiol in starved MLO-Y4 osteocytes. In these cells, a relationship between oxidative stress-induced apoptosis and up-regulation of active FGF23 levels due to MAP Kinases activation with involvement of the transcriptional factor (NF-kB) has been demonstrated. The active FGF23 increase can be due to up-regulation of its expression and post-transcriptional modifications. 17β–estradiol prevents the increase of FGF23 by inhibiting JNK and NF-kB activation, osteocyte apoptosis and by the down-regulation of osteoclastogenic factors, such as sclerostin. No alteration in the levels of dentin matrix protein 1, a FGF23 negative regulator, has been determined. The results of this study identify biological targets on which drugs and estrogen may act to control active FGF23 levels in oxidative stress-related bone and non-bone inflammatory diseases.

## 1. Introduction

Fibroblast growth factor 23 (FGF23), a phosphaturic hormone produced primarily by osteocytes and osteoblasts [1,2,3], plays an important role in the regulation of phosphate (Pi) homeostasis and bone mineralization [4]. The main target organ of FGF23 activity is the kidney, through its signaling receptor and co-receptor α-Klotho in the proximal renal tubules [5]. FGF23 down-regulates the expression of the sodium phosphate co-transporter, stimulating the release of Pi in the urine, and preventing the 1α-hydroxylation of 25-hydroxyvitamin D (25-(OH)D) with consequent decrease in the absorption of calcium and Pi by the intestine [6]. Altogether these changes cause decrease of blood Pi and 1-25-(OH)D levels, and consequently the activation of FGF23 secretion both in vivo and in vitro [7,8]. The intact circulating form of FGF23 (32–35 KDa) is the biologically active molecule (active FGF23) which undergoes mucin-type O-glycosylation in the Golgi apparatus of osteocytes by the enzyme *N*-acetyl galactosaminyltransferase 3 (GalNT3). This is required for the secretion of active FGF23 by blocking its proteolytic cleavage by the protease furin. This proteolytic process is responsible for formation of the inactive FGF23 c-terminal form (15–17 KDa) [9,10]. Osteocytes are the primary source of FGF23 and other factors related to its action and regulation, such as phosphate regulating endopeptidase homolog X-linked (PHEX), dentin matrix protein 1 (DMP1), matrix extracellular phosphoglycoprotein (MEPE), and sclerostin [11,12]. Moreover, osteocytes release cytokines and signaling molecules, such as receptor activator of nuclear factor kappa-Β ligand (RANKL) and tumor necrosis factor-α (TNFα), which in paracrine and endocrine fashion, similar to FGF23, playing a crucial role not only in the pathophysiological processes involving bone but also other targets, such as kidney, heart and immunological and vascular systems [13,14]. Systemic and local factors, including levels of the inorganic Pi and pyrophosphate molecules, regulate FGF23, which is involved locally in osteocyte regulatory activity in bone remodeling and mineralization processes [15,16]. A reduction of active FGF23 induces hyperphosphatemia and up-regulates genes involved in osteoblast differentiation and matrix mineralization, contributing to the development of calcification [17,18]. On the other hand, an increase of active FGF23 is related to hypophosphatemic pathologies such as X-linked hypophosphatemic rickets and tumor-induced osteomalacia [19].

Many data relate high levels of reactive oxygen species (ROS) to defective bone remodeling and mineralization processes regulated by osteoblast and osteoclast activity [20,21,22,23,24], and controlled by osteocytes, through secretion of factors that can modulate both bone formation and resorption [25,26,27]. It has also been demonstrated that high levels of ROS induce marked apoptosis in osteoblasts and osteocytes [27,28,29,30], and both oxidative stress and osteocyte apoptosis are linked to deficiency of estrogens, the aging process and chronic glucocorticoid treatment with consequent decrease in bone mineral density [15,31,32]. In fact, ovariectomy increases osteocyte apoptosis, leading to the accumulation of bone microdamage unable to be repaired through the bone remodeling process, with consequent abnormal activation of osteoclast activity [33,34,35]. Estrogen loss alters osteoblast activity and reduces mineralization processes, inducing bone resorption and osteoporosis, thereby deteriorating bone microarchitecture, which results in reduced mechanical strength and increased risk of fracture [36,37]. Recent, studies have shown that, in bone and inflammatory diseases, high levels of ROS are related to increased FGF23 expression leading to imbalance of the calcium-phosphate metabolism and alteration of the mineralization processes [14,38,39,40]. Previously, we demonstrated a clear relationship between apoptosis, induced by high levels of ROS, and an increase in osteoclastogenic factors, such as RANKL and sclerostin [30,40], in osteocytes as well as a reduction in the mineralization process of osteoblasts in the presence of oxidative stress [22,24]. In vivo and in vitro data demonstrate that natural antioxidants prevent and/or revert the negative effects of oxidative stress on bone tissue through the maintenance of osteocyte activity, activation of osteoblast differentiation and mineralization processes [20,22,24,30,40,41]. In fact, the protective effects of estrogens on bone seem to be due to their ability to attenuate oxidative stress [42]; however, we previously showed that estrogen prevents oxidative stress-induced apoptosis and the increase of osteoclastogenic factors in MLO-Y4 cells by a redox-independent process [43]. To our knowledge there are no data that relate oxidative stress-induced apoptosis (OSIA) of osteocytes to alterations of active FGF23 levels. Similarly, there are few data on the molecular processes and signaling pathways through which estrogens can modulate the intracellular levels of active FGF23 and its release in the presence of oxidative stress in osteocytes. FGF23 is controlled both locally and systemically through the action of various hormones and factors, but the molecular mechanisms related to its transcriptional and post-transcriptional regulation in osteocytes are still not completely understood.

The aim of this study was to identify alterations of active FGF23 intra- and extracellular levels in osteocytes that have undergone apoptosis induced by oxidative stress and the protective role of 17-β-estradiol (17β–E2) in maintaining normal levels of this phosphaturic hormone. The involvement of molecular mechanisms and factors related to bone remodeling and mineralization processes through which 17β–E2 can act on active FGF23 was also investigated. The present study was performed in MLO-Y4, a murine osteocyte-like cell line, which is considered a valid in vitro model to study the signals generated by viable or apoptotic osteocytes, in order to regulate the remodeling process in response to microdamage and bone disease [44]. The culture of cells in serum-free medium (starvation) causes OSIA in MLO-Y4 [43], and this in vitro method mimics an in vivo metabolic condition that occurs in the bone environment after microdamage and/or lack of various endocrine factors, including estrogens [45]. Therefore, starved MLO-Y4 cells constitute a study model to characterize regulation mechanisms and specific factors involved in osteocyte activity in the presence of oxidative stress and abnormal apoptosis. This study demonstrates, in murine osteocytic cells, the relationship between OSIA and the up-regulation of active FGF23, which 17β–E2 is able to prevent by inhibiting the activation of mediators involved in apoptotic processes.

## 2. Results

### 2.1. Effect of OSIA on Intra- and Extracellular Levels of Active FGF23 in Starved MLO-Y4 Cells

**Figure 1:**
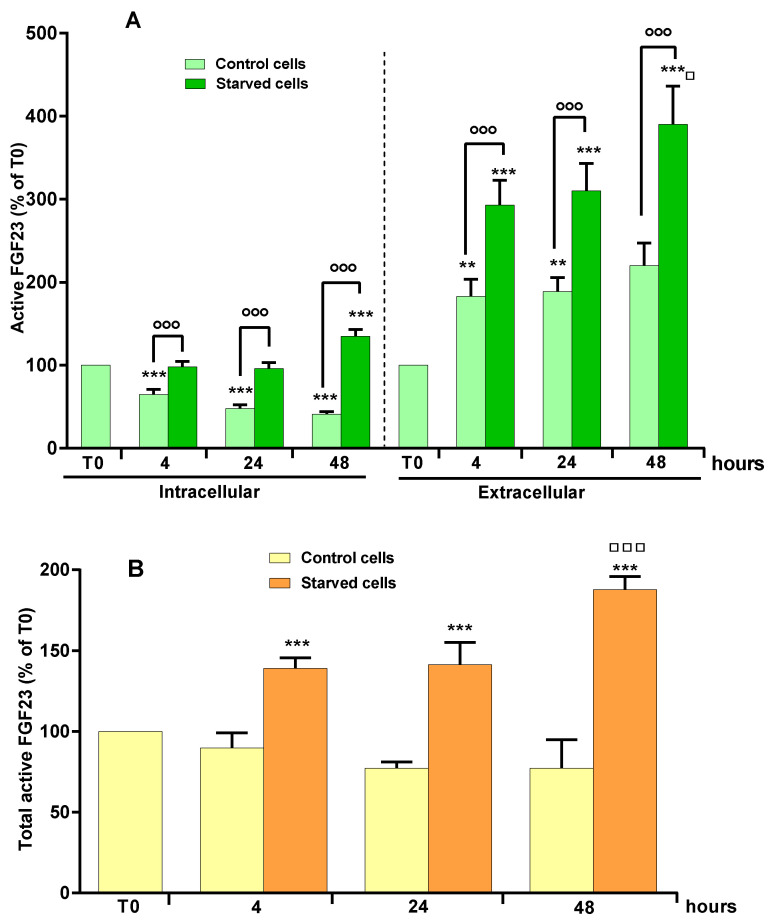
Intracellular, extracellular, and total levels of active FGF23 in starved MLO-Y4 cells in the presence of OSIA. (**A**) Active FGF23 levels were assayed using ELISA kit in cell lysates (intracellular) and in the respective culture media (extracellular) of MLO-Y4 cells cultured for 4, 24 and 48 h in complete medium (control cells) and in serum-free medium (starved cells), as reported in Materials and Methods. (**B**) Total levels of active FGF23 are reported as the sum of intracellular and extracellular levels. The data, expressed as percentage relative to the starting time T0, are reported as the mean ± SD of four replicate experiments. ** *p* ≤ 0.01; *** *p* ≤ 0.001 compared to T0; ◦◦◦ *p* ≤ 0.001 compared to the respective control cells; ^□^ *p* ≤ 0.05; ^□□□^ *p* ≤ 0.001 compared to 4- and 24-h-starved cells.

The intra- and extracellular levels of active FGF23 were measured in cell lysates and in the culture media, in complete medium (control) and in starvation medium after 4, 24 and 48 h. Under these experimental conditions, starvation induces oxidative stress and consequent apoptosis as previously demonstrated [30,43]. Figure 1A,B reports the values of active FGF23 measured as a percentage compared to starting time T0 in complete medium.

Figure 1A shows a significant decrease in intracellular levels of active FGF23 after 4, 24 and 48 h in lysates of control cells cultured in complete medium. This behavior was related to the respective extracellular increase of active FGF23 levels at all studied times (Figure 1A). In starved cells, the levels of intracellular active FGF23 at 4 and 24 h were similar to those measured at T0, and significantly higher than those of the respective control cells (Figure 1A). The extracellular FGF23 levels in starved cells also increased at various study times (Figure 1A). The maximum increase in intra- and extracellular levels of active FGF23 was observed after 48 h of starvation (Figure 1A). Figure 1B shows that, in control cells, the total values of active FGF23, obtained by the sum of the intra- and extracellular levels, were similar to T0, as opposed to what occurs in starved cells, in which total FGF23 increased significantly over time. 

### 2.2. Role of 17β–E2 on OSIA-Induced Intra- and Extracellular Levels of Active FGF23 in Starved MLO-Y4 Cells 

**Figure 2:**
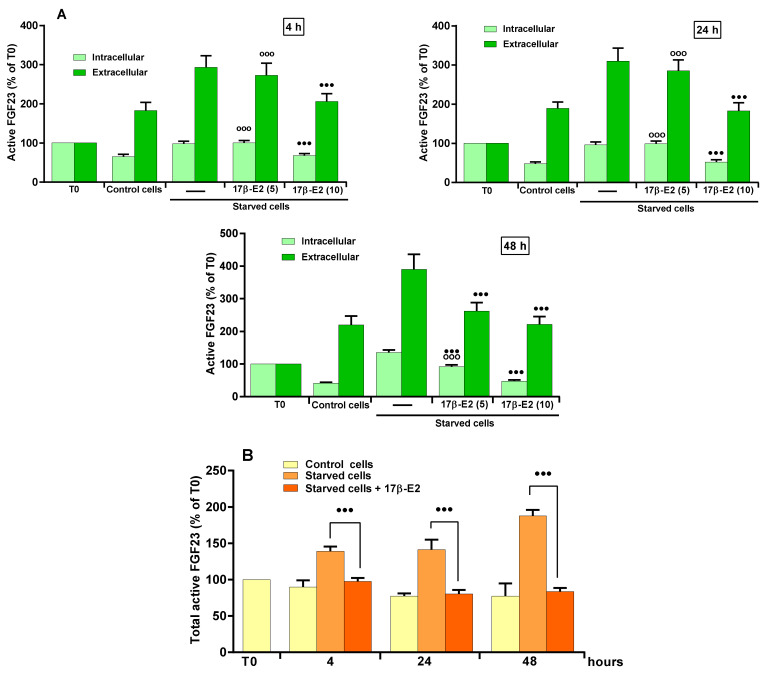
17β-E2 effect on active FGF23 levels in MLO-Y4 cells in the presence of OSIA. (**A**) Active FGF23 levels were assayed using ELISA kit in cell lysates (intracellular) and in the respective culture medium (extracellular) of MLO-Y4 cells cultured for 4, 24 and 48 h in complete medium (control cells) and in serum-free medium (starved cells). Starved cells were treated or not with 5 or 10 nM 17β-E2, as reported in Materials and Methods. (**B**) Total levels of active FGF23 are reported as the sum of intracellular and extracellular levels, and starved cells were treated or not with 10 nM 17β-E2. The data, expressed as percentage relative to the starting time T0, are reported as the mean ± SD of four replicate experiments. ◦◦◦ *p* ≤ 0.001 compared to the respective control cells; ^●●●^ *p* ≤ 0.001 compared to the respective untreated and starved cells.

The concentrations (5 and 10 nM) used for studying the role of 17β–E2 on OSIA-induced increase in active FGF23 levels were those able to prevent apoptosis due to oxidative stress in MLO-Y4 cells [43]. Figure 2A shows that the pre-treatment of starved MLO-Y4 cells with 5 nM 17β–E2 did not affect the increase of the intra- and extracellular levels of active FGF23 after 4 and 24 h of starvation. 

However, at this concentration, 17β–E2 prevented the extra- and intracellular FGF23 level increase, partially or totally, respectively, only after 48 h of starvation. On the contrary, 10 nM 17β–E2 was more effective and prevented the increase of both intra- and extracellular levels at all times (Figure 2A). The ability of 10 nM 17β–E2 was also evident on total active FGF23 levels (Figure 2B).

### 2.3. Role of MAP Kinases on OSIA-Induced Intra- and Extracellular Levels of Active FGF23 in Starved MLO-Y4 Cells 

**Figure 3:**
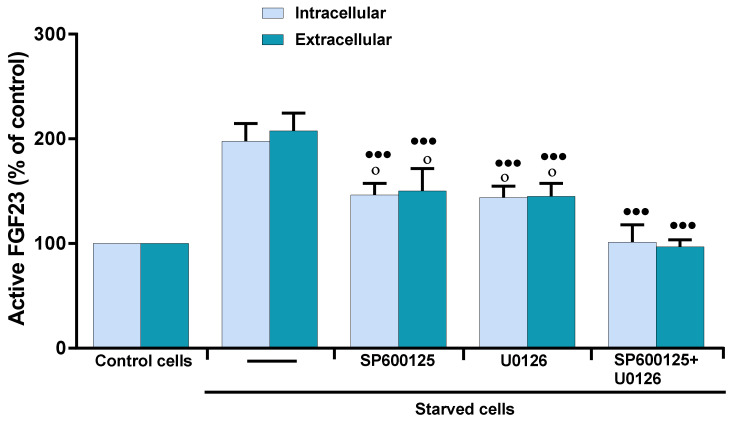
Effect of U0126, SP600125 on active FGF23 levels in starved MLO-Y4 cells in the presence of OSIA. Active FGF23 levels were assayed using ELISA kit in cell lysates (intracellular) and in the respective culture medium (extracellular) of MLO-Y4 cells cultured for 24 h in complete medium (control cells) and in serum-free medium (starved cells). Starved cells were treated or not with 5 µM U0126 or 25 µM SP600125 or both, as reported in Materials and Methods. The data, expressed as percentage relative to the control values, are reported as the mean ± SD of four replicate experiments. ◦ *p* ≤ 0.05 compared to the respective control cells; ^●●●^ *p* ≤ 0.001 compared to the respective untreated and starved cells.

Previously, we demonstrated in apoptotic MLO-Y4 osteocytes that oxidative stress activates c-Jun N-terminal kinase (JNK) and extracellular signal-regulated kinase (ERK1/2), and that 17β–E2 is only able to prevent the activation of JNK, a kinase mainly involved in the induction of osteocyte apoptosis due to oxidative stress [30]. Therefore, the effect of JNK and ERK1/2 on the increased intra- and extracellular levels of active FGF23 was evaluated by using specific inhibitors at concentrations capable of inhibiting the activation of these kinases in MLO-Y4 cells starved for 24 h [30]. Figure 3 shows that SP600125 and U0126, inhibitors of JNK and ERK1/2, respectively, significantly but partially inhibited the increase of intra- and extracellular levels of active FGF23.

In fact, the values obtained were significantly higher than those of control. The totally inhibition of active FGF23 increase occurred when the cells were treated simultaneously with the inhibitors of both kinases. This indicates that both JNK and ERK1/2 can be involved in active FGF23 increase in starved MLO-Y4 cells and suggests that the effect of 17β–E2, in maintaining FGF23 levels at the control values, is due to its ability to inhibit these kinases and/or factors downstream from their signaling pathway.

### 2.4. Role of NF-kB on 17β–E2 Effect on OSIA-Induced Intra- and Extracellular Levels of Active FGF23 in Starved MLO-Y4 Cells

**Figure 4:**
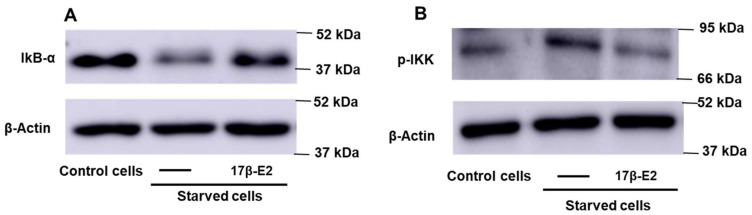
IkB-α and p-IKK levels in starved MLO-Y4 cells treated or not with 17β-E2 in the presence of OSIA. MLO-Y4 cells were cultured for 1 h in complete medium (control cells) and in serum-free medium (starved cells). Starved cells were treated or not with 10 nM 17β-E2, as reported in Materials and Methods. (**A**) IkB-α and (**B**) p-IKK levels were detected in cell lysates by Western blot analysis using anti-IkB-α or anti-p-IKK.

Nuclear factor kappa B (NF-κB) is one of the transcription factors activated by ERK1/2 and involved in bone loss following estrogen withdrawal and in FGF23 regulation [46,47,48,49]. The activation of NF-κB is due to the phosphorylation of the IKK kinase, which phosphorylates and inhibits IKB-α, the endogenous inhibitor of NF-κB, by promoting its degradation. NF-κB can thus move to the nucleus and activate the transcription of numerous genes. To evaluate the activation of NF-κB in apoptotic osteocytes, the levels of phosphorylated IKK (pIKK) and IkB-α were measured, in the absence and in the presence of 10 nM 17β-E2 at 1 h from the MLO-Y4 starvation. Figure 4A,B shows a decrease in IkB-α and an increase in p-IKK levels, indicating that NF-κB can be activated in starved MLO-Y4 cells. 

**Figure 5:**
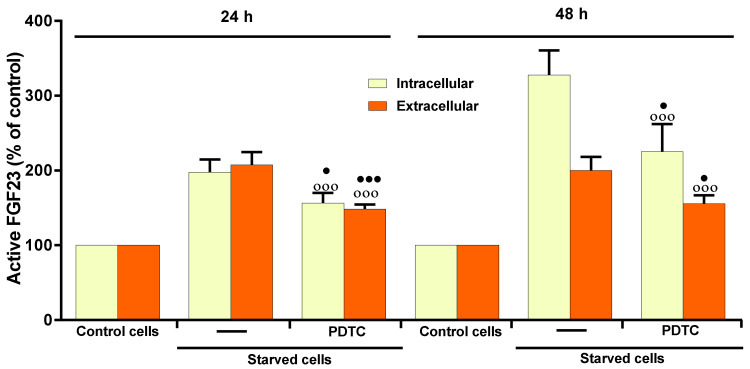
Effect of PDTC on active FGF23 levels in starved MLO-Y4 cells in the presence of OSIA. Active FGF23 levels were assayed using ELISA kit in cell lysates (intracellular) and in the respective culture medium (extracellular) of MLO-Y4 cells cultured for 24 h and 48 h in complete medium (control cells) and in serum-free medium (starved cells). Starved cells were treated or not with 50 µM PDTC, as reported in Materials and Methods. The data, expressed as percentage relative to the control values, are reported as the mean ± SD of four replicate experiments. ◦◦◦ *p* ≤ 0.001 compared to the respective control cells. ^●^ *p* ≤ 0.05; ^●●●^ *p* ≤ 0.001 compared to the respective untreated and starved cells.

17β–E2 prevented the activation of NF-κB in MLO-Y4 cells; in fact, the bands of p-IKK and IkB-α were similar to those of their controls (Figure 4A,B). Figure 5 shows that the pre-treatment of MLO-Y4 with 50 µM pyrrolidine dithiocarbamate (PDTC), a specific inhibitor of NF-κB, inhibited the intra- and extracellular levels of active FGF23 after 24 and 48 h of starvation, indicating that this transcriptional factor is effectively involved in the increase of active FGF23 in starved MLO-Y4. However, PDTC was unable to totally prevent the increase of FGF23, as observed with MAPK inhibitors (Figure 5).

### 2.5. Effect of 17β–E2 on DMP1 Levels and Relationship with the Levels of Active FGF23 in the Presence of OSIA in Starved MLO-Y4 Cells

**Figure 6:**
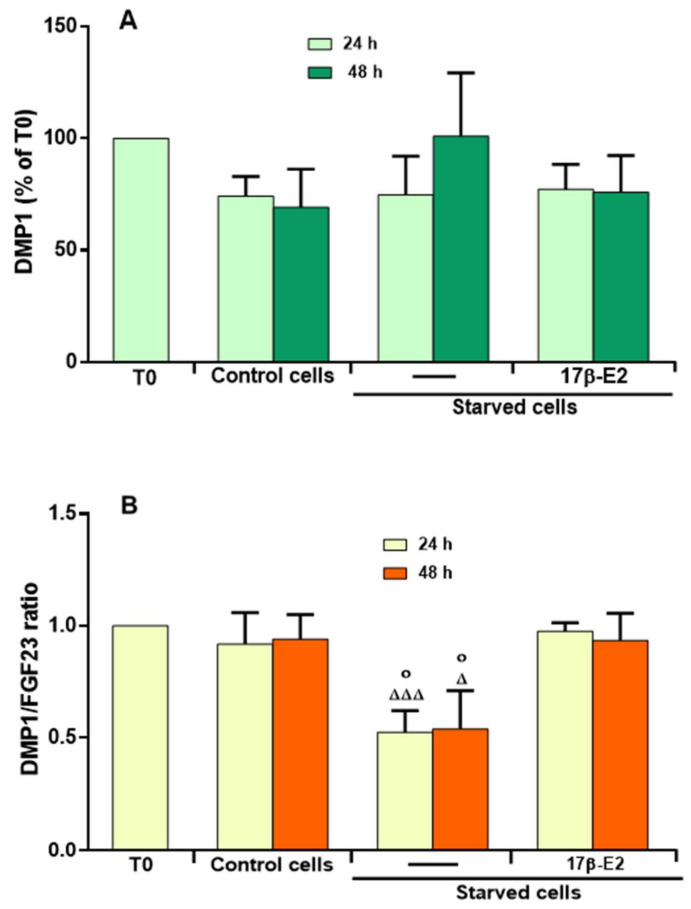
Levels of intracellular DMP1 in starved MLO-Y4 cells treated or not with 17β-E2 and DMP1/FGF23 ratio in the presence of OSIA. (**A**) DMP1 levels were assayed using ELISA kit in cell lysates of MLO-Y4 cells cultured for 24 and 48 h in complete medium (control cells) and in serum-free medium (starved cells). Starved cells were treated or not with 10 nM 17β-E2, as reported in Materials and Methods. (**B**) Values of the ratio were measured between total levels of DMP1 and active FGF23. DMP1 and DMP1/FGF23 ratio levels are expressed as percentage or fold-increase compared to value obtained at starting time T0. The data are the mean ± SD of four replicate experiments. ◦ *p* ≤ 0.05 compared to the respective control cells; ^Δ^ *p* ≤ 0.05; ^Δ^^ΔΔ^ *p* ≤ 0.001 compared to the respective starved and 17β-E2-treated cells.

Since DMP1 is a factor produced by osteocytes and closely related to FGF23 regulation and mineralization processes [1], the intra- and extracellular levels of this protein were evaluated in MLO-Y4 cells starved for 24 and 48 h and treated or not with 10 nM 17β-E2. We were able to measure intracellular DMPI levels in cell lysates, while no extracellular value was detected under these experimental conditions. This may be due to the presence of very low extracellular levels of DMP1 below the sensitivity of the assaying method; therefore, the values measured in the cell lysates are considered the total values of DMP1. Figure 6A shows that the values of DMP1, measured as percentage of the level obtained at starting time T0, did not change significantly in starved cells treated or not with 17β–E2 as compared to control cells. 

Given that the relationship between the levels of DMP1 and FGF23 is important for the osteogenesis and mineralization process [50], we measured the ratio between the total levels of these two factors. Fig 6B shows that, in starvation, DMP1/FGF23 ratio decreased significantly at 24 and 48 h compared to respective controls and that 17β–E2 prevented this imbalance.

**Figure 7:**
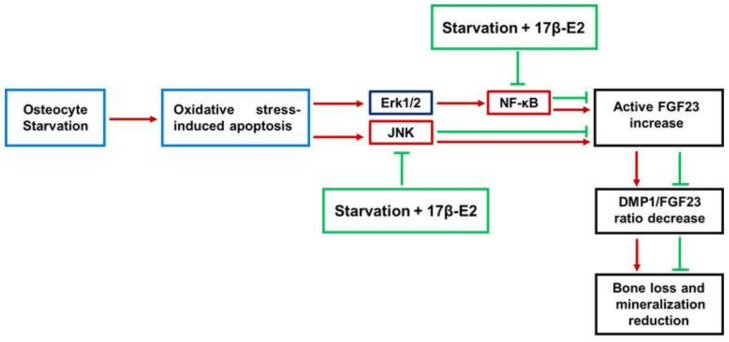
Summary of the possible mediators involved in the up-regulation of the active FGF23 levels in oxidative stress-induced apoptosis in starved MLO-Y4 cells and protective role of 17β-E2.

Finally, Figure 7 summarizes the possible mediators involved in the regulation of the active FGF23 levels by 17β-E2 in the presence of OSIA in starved MLO-Y4 cells. It is evident that JNK and ERK1/2 activation induces the up-regulation of active FGF23. However, the effect of ERK1/2 is mediated by NF-κB activation. 

Also, it is shown that, through the inhibition of these factors, 17β-E2 is able to maintain normal levels of active FGF23 and DMP1/FGF23 ratio.

## 3. Discussion

This study uncovers novel mechanisms, involving JNK and ERK pathways, through which OSIA influences the intra- and extracellular levels of the intact active FGF23 hormone in osteocytes [4,5]. In particular, the oxidative stress condition induces the apoptosis and the activation of JNK and ERK1/2 in starved MLO-Y4 cells [43], and the contemporary inhibition of these pathways prevents the up-regulation of active FGF23 levels detected in these cells. Moreover, 17β-E2 exerts a protective role through the maintenance of normal levels of active FGF23 in the presence of high levels of ROS. An up-regulation of total active FGF23 level (extra + intra) is present in starved MLO-Y4 cells versus control cells, suggesting that the up-regulation of FGF23 is due to its increased expression. Over time, the trend of intracellular content of active FGF23 in starved cells differs from that of control cells in which a quick decrease is observed after 4 h due to its release in the extracellular medium. In starved cells, intracellular FGF23 increases over time, as well as its release; the highest levels of active FGF23 were reached after 48 h. This suggests a different intracellular regulation of active FGF23 in starved cells, in which its up-regulation may be related not only to activation of its expression, but also to the mechanisms that regulate its release. The latter include an increased expression of glycosyltransferase GALNT3 and/or inhibition of furin convertase activity with an increase of FGF23 glycosylation and/or a reduction of the proteolytic cut of intact FGF23, respectively. In fact, serum FGF23 levels are strongly regulated by FGF23 transcription and activity of these enzymes which can be controlled by pro-inflammatory mediators and iron deficiency related to chronic and acute inflammation [49,51,52]. 

In the present study, the up-regulation of active FGF23 seems to be related to the increase of apoptosis and ROS levels in starved MLO-Y4 cells [30,40,43]. These findings confirm what has been observed in the rat osteoblastic cell line, UMR-106, regarding the increased expression of FGF23 due to high concentrations of Pi, which stimulates the production of high levels of ROS [38]. Moreover, Pi induces the maximum expression of FGF23 after 72 h indicating that phosphate regulation seems to have a long time frame. H_2_O_2_ treatment, also, significantly increases FGF23 expression after 48 h in UMR-106 cells, and this appears to be similar to what we observed in starved MLO-Y4 cells [38]. These data also agree with the oxidative stress status, osteocyte apoptosis and the high levels of FGF23 in chronic kidney disease (CKD) [53,54,55], as well as the increase of apoptotic osteocytes related to the enhancement of FGF23 in glucocorticoid-induced osteoporosis, with consequent decrease in mineralization processes [39]. In contrast, the down-regulation of oxidative stress inhibits the expression of FGF23 in the femur and helps to balance the calcium-phosphate metabolism [56]. This confirms that a link between OSIA and the up-regulation of active FGF23 is present in starved MLO-Y4 cells. 

Several studies have shown that estrogen deficiency and bone inflammatory pathologies, through increased ROS production, can cause apoptosis and alter osteocyte function with consequent up-regulation of factors, such as sclerostin, RANKL, TNFα, FGF23, and other signaling molecules involved in bone pathologies, such as osteoporosis, and in inflammatory diseases of other organs [14,39,57]. Estrogen treatment prevents osteoblast and osteocyte apoptosis, allowing the maintenance of normal bone remodeling and mineralization [43,57,58]. In fact, 10 nM 17β–E2 reduces apoptosis in starved MLO-Y4 cells, inhibiting JNK activation through a non-redox regulated mechanism, therefore reducing the levels of active FGF23. In particular, after 4 h, 17β–E2 is already able to prevent the increase of active FGF23. Previously, under the same experimental conditions, it has been demonstrated that 17β–E2 determines its maximum anti-apoptotic and anti-osteoclastogenic effect without diminishing ROS levels [43]. In addition, in starved MLO-Y4 cells, it has been demonstrated that 10 nM 17β–E2 shows a weak antioxidant effect only after 24 h, not due to a direct ROS scavenger effect, but mostly due to its ability to up-regulate antioxidant enzymes which requires longer times [43,59,60,61]. For this reason, 17β–E2 does not prevents early apoptosis and up-regulation of active FGF23 through its antioxidant ability, which, however, can contribute to the normalization of FGF23 levels only after 48 h. Therefore, 17β–E2 can immediately contribute to maintenance of normal bone metabolism rate through non-redox-regulated mechanisms, even in the presence of oxidative stress. 

The up-regulation of active FGF23 is also due to the activation of ERK1/2 in starved MLO-Y4 cells, and this agrees with the involvement of these kinases in the up-regulation of FGF23 and GALNT3 expression in UMR-106 cells stimulated with high Pi concentrations [38,62]. In starved MLO-Y4 cells, oxidative stress induces both ERK1/2 and JNK activation; however, 17β–E2 is able to inhibit only JNK activation and not that of ERK1/2 [30,43]. Nevertheless, 17β–E2 totally prevents the up-regulation of FGF23, similar to that verified with simultaneous inhibition of both kinases. This may be due to the ability of 17β–E2 to inhibit JNK activation [43], and transcriptional factors downstream from ERK1/2 activation. The activation of NF-κB has been demonstrated in starved MLO-Y4 cells along with its involvement in the up-regulation of active FGF23 and inhibition by 17β-E2. In fact, 17β–E2 inhibits NF-kB by preventing its pro-inflammatory-factors-induced activation and nuclear translocation in various cell lines [63,64] as well as NF-kB expression in a human osteoblastic cell line, MG-63 [65]. NF-kB is also a well-known regulator of FGF23 expression [8,66], and it is involved in calcium-up-regulated FGF23 expression in UMR106 cells [67]. Moreover, the activation of NF-κB, due to oxidative stress-induced ERK1/2 activation, negatively regulates osteoblast differentiation and mineralization processes [24,68,69], and induces osteoclast differentiation [70]. Inflammation is one of the major inducers of FGF23 [51], and apoptotic osteocytes produce pro-inflammatory cytokines, such as TNFα, which stimulate FGF23 production by NF-kB dependent mechanisms in the osteocyte-like cell line IDG-SW3 [49]. TNFα induces osteocyte apoptosis through NF-kB activation contributing to osteoporosis in postmenopausal women, and it induces osteocyte apoptosis and FGF23 increase in glucocorticoid-induced osteoporosis [39,71,72]. For this reason, we speculate that, in apoptotic MLO-Y4 a production of TNFα can also occur, and this may be involved in part in the increase of FGF23 through ERK-dependent NF-κB activation [73]. Overall, these data confirm the involvement of NF-kB in OSIA-induced FGF23 upregulation, and that 17β-E2 down-regulates active FGF23 levels by inactivating this transcriptional factor, likely through a non-redox-regulate mechanism in MLO-Y4 starved cells. 

17β–E2 is able to prevent OSIA-induced up-regulation of osteoclastogenic factors, such as RANKL and sclerostin, and the increase of RANKL/OPG ratio in starved MLO-Y4 cells [43]. These events are related in vivo with estrogen deficiency, bone microdamage and bone loss through activation of osteoclastogenesis and alteration of the remodeling process [20]. In particular, sclerostin, a well-known inhibitor of Wnt-signaling and the osteogenic process, alters the concentrations of hormones, such as vitamin D metabolites and FGF23, that regulate bone mineralization [14,74]. In fact, a positive association between high serum levels of sclerostin and FGF23 has also been found in CKD and other bone disorders related to abnormalities in mineral metabolism [14,75], and inactivating mutations in SOST, the gene encoding sclerostin, are associated with low levels of FGF23, overgrowth, and sclerosis of the skeleton [76]. For this reason, it is possible that also in starved MLO-Y4 cells the up-regulation of FGF23 may be related to high sclerostin levels and the inhibition of mineralization processes [30,43,77]. Therefore, the ability of 17β–E2 to prevent the up-regulation of active FGF23 may agree with its capacity to inhibit the increase of osteoclastogenic factors [43]. Some data suggest that sclerostin indirectly up-regulates FGF23 gene expression by inhibiting factors produced by osteocytes, such as DMP1 and PHEX, negative regulators of FGF23, that are involved in phosphate homeostasis and mineralization [75,78,79]. In particular, DMP1 prevents FGF23 increase [55], and its levels seem to be inversely related to those of active FGF23 [1]. However, in the present study, no variation in DMP1 levels in the presence or absence of 17β–E2 has been determined showing that, in starved MLO-Y4 cells, DMP1 levels are not inversely related to those of active FGF23. This suggests that different signaling may be involved in the increase of OSIA-induced FGF23 in starved MLO-Y4 cells. In fact, data in the literature has shown that an increase in GALNT3 levels favors the increase of active FGF23 levels through glycosylation, in response to pro-inflammatory factors, and that the activation of kinases by FGF23 phosphorylation increases its storage and release [49,80]. In vivo studies have also demonstrated that the levels of serum intact FGF23 are mainly regulated by post-transcriptional mechanisms, and, together with other factors produced by osteocytes and other districts, contribute to maintain Pi homeostasis and the mineralization process [49]. Therefore, unlike what other researchers have observed, OSIA does not up-regulate FGF23 levels by down-regulating DMP1 in starved MLO-Y4 cells and the preventive action of 17β–E2 in maintaining FGF23 normal levels is not related to DMP1 levels.

## 4. Materials and Methods

### 4.1. Cell Culture and Treatment

MLO-Y4 osteocyte-like cells (a gift from Dr. Lynda Bonewald, University of Missouri-Kansas City) were cultured at 37 °C in a 5% CO_2_ humidified atmosphere to 70–80% confluence in alpha-MEM medium supplemented with 5% Calf Serum (HyClone, GE Healthcare, Chicago, IL, USA), 5% Fetal Bovine Serum (HyClone, GE Healthcare), 2 mM L-glutamine, 72 mg/l penicillin and 100 mg/mL streptomycin (complete medium, CM). Subsequently, this medium was removed and MLO-Y4 were grown for 30 min in a medium in which FBS was substituted with charcoal stripped FBS (Sigma-Aldrich, St. Louis, MO, USA). Then, cells at starting point (T0) were immediately collected whereas other cells were cultured for another 4, 24 or 48 h in fresh medium containing 10% charcoal-stripped FBS (C, control cells) or in serum free medium (S, starved cells) in the presence or not of 5 or 10 nM 17β–E2 (Sigma-Aldrich). 0.01% ethanol (vehicle for 17β–E2) was added to all untreated 17β–E2 cells. For experiments with inhibitors, cells were pretreated for 30 min in complete medium in the presence or not of 25 μM SP 600125 (JNK1/2 inhibitor), 5 μM U0126 (ERK1/2 inhibitor), or 50 μM PDTC (NF-κB inhibitor) (Sigma-Aldrich). After removal of this medium MLO-Y4 cells were cultured for 24 and/or 48 h in complete medium or in starved medium in the presence or not of the inhibitors. The inhibitor concentrations used are those that induced the maximum inhibition without interfering with cellular viability [30,81].

### 4.2. FGF23 and DMP1 Assay

Active FGF23 and DMP1 intracellular and extracellular levels were evaluated in cell lysates and culture media using mouse FGF23 ELISA kit and DMP1 ELISA Kit (Abcam, Cambridge, UK), respectively, according to the manufacturer’s instructions. MLO-Y4 cells were seeded in 12-well plates and treated with 17β–E2 or inhibitors for 4, 24 or 48 h, as described above. Cell lysates were performed detaching and collecting MLO-Y4 by centrifugation at 130× *g* for 10 min. Cells, washed in cold phosphate buffer saline (PBS), were suspended in PBS and sonicated four times. Then, cell lysates were centrifuged at 1500× *g* for 10 min at 4 °C and FGF23 and DMP1 assays were performed in the supernatants. Absorbance was measured at 450 nm with iMarkTM Microplate Absorbance Reader (BioRad, Hercules, CA, USA). Data were normalized on total protein content and FGF23 and DMP1 levels were expressed as a percentage of the respective levels measured in control set as 100%. 

### 4.3. Western Blot Analysis 

IKB-α and phosphorylated IKK levels were performed by Western blot in MLO-Y4 seeded in 60 mm tissue culture dishes and treated for 1 h with or without 17β–E2, as reported above. Cells lysed for 30 min at 4 °C in ice-cold RIPA buffer (50 mM Tris/HCl pH 7.5, 1% Triton X100, 150 mM NaCl, 100 mM NaF, 2 mM EGTA, phosphatase and protease inhibitor cocktails, Sigma-Aldrich) were centrifuged at 11,600 *g* for 10 min. Supernatants were collected and protein concentrations were determined by the bicinchoninic acid solution protein reagent assay (Pierce Biotechnology, Waltham, MA, USA) [82] using bovine serum albumin as standard (Sigma-Aldrich). Equal amounts of total proteins (40–60 μg) were loaded in each line and were subjected to sodium dodecyl sulphate-polyacrylamide gel electrophoresis (SDS/PAGE) on a 10% gel and subsequently electrotransferred to PVDF membrane (GE Healthcare). Membrane was probed with specific primary monoclonal antibodies anti-phospho-IKKα/β or anti-IkB-α (dilution 1:1000; #2647; #4812; Cell Signalling Technology, Danvers, MA, USA) or anti-β-actin (dilution 1:1000; sc-47778; Santa Cruz Biotechnology, Dallas, TX, USA). Secondary antibodies conjugated to horseradish peroxidase (dilution 1:1000; anti-rabbit sc-2357, anti-goat sc-2354; Santa Cruz Biotechnology) were used to detect antigen-antibody complexes using a chemiluminescence reagent kit (Clarity Western ECL Substrate, Bio-Rad). Digital images of bands were detected by Amersham Imager A600 (GE Healthcare), and densitometric analysis, normalized with β-actin band, was performed using Image J software (National Institutes of Health, Bethesda, MD, USA). Values were expressed as percentage of the control set as 100%.

### 4.4. Statistical Analysis

Each experiment was performed a minimum of three times. Data are expressed as means ± SD and statistical significance was determined by one-way ANOVA analysis with Bonferroni’s multiple comparison test, using the GraphPad Prism Software (California, USA, San Diego). *p* ≤ 0.05 was considered statistically significant.

## 5. Conclusions

The novel data in the present study show a relationship between OSIA and the up-regulation of the intra- and extracellular levels of active FGF23 due to activation of both JNK and ERK1/2 signaling pathways, with the involvement of NF-κB, in starved MLO-Y4 cells. 17β–E2 inhibits quickly and in the presence of oxidative stress the osteocyte apoptosis and activation of osteoclastogenic factors [43], with consequent active FGF23 decrease. OSIA does not affect the levels of DMP1, a negative regulator of FGF23, in the presence or absence of 17β–E2. Therefore, 17β–E2 maintains the normal levels of DMP1/FGF23 ratio and ensures a normal osteogenic process by preventing FGF23 up-regulation. Overall, these in vitro results identify JNK and ERK as biological targets on which specific drugs and 17β–E2 can act in the presence of oxidative stress to maintain normal bone remodeling and mineralization processes.

## Data Availability

The data presented in this study are available on request from the corresponding author.
